# A G-Fresnel Optical Device and Image Processing Based Miniature Spectrometer for Mechanoluminescence Sensor Applications

**DOI:** 10.3390/s19163528

**Published:** 2019-08-12

**Authors:** Kyung-Pyo Min, Jaehwan Kim, Kyo D. Song, Gi-Woo Kim

**Affiliations:** 1 Department of Mechanical Engineering, Inha University, Incheon 22212, Korea; 2 Department of Engineering, Norfolk State University, Norfolk, VA 23504, USA

**Keywords:** G-Fresnel optical device, miniature spectrometer, digital image processing, HSV color space, mechanoluminescence sensor

## Abstract

This paper presents a miniature spectrometer fabricated based on a G-Fresnel optical device (i.e., diffraction grating and Fresnel lens) and operated by an image-processing algorithm, with an emphasis on the color space conversion in the range of visible light. The miniature spectrometer will be cost-effective and consists of a compact G-Fresnel optical device, which diffuses mixed visible light into the spectral image and a μ-processor platform embedded with an image-processing algorithm. The RGB color space commonly used in the image signal from a complementary metal–oxide–semiconductor (CMOS)-type image sensor is converted into the HSV color space, which is one of the most common methods to express color as a numeric value using hue (*H*), saturation (*S*), and value (*V*) via the color space conversion algorithm. Because the HSV color space has the advantages of expressing not only the three primary colors of light as the *H* but also its intensity as the *V*, it was possible to obtain both the wavelength and intensity information of the visible light from its spectral image. This miniature spectrometer yielded nonlinear sensitivity of hue in terms of wavelength. In this study, we introduce the potential of the G-Fresnel optical device, which is a miniature spectrometer, and demonstrated by measurement of the mechanoluminescence (ML) spectrum as a proof of concept.

## 1. Introduction

Spectrophotometers have been used to analyze the spectral information (wavelength and intensity) of fluorescence from particular substances in the area of biology, biomedicine, chemistry and astronomy. The optical properties of substances can be drawn from light transmitted or absorbed from the substances and the spectrum provides signature information about their physical and chemical properties [1,2]. For instance, bio-medical sensors based on spectrophotometry can detect and monitor the biological condition of the human body to diagnose disease and analyze the concentration and pH of chemical and biological materials [3,4,5,6,7]. Spectrophotometers commonly use a miniature spectrometer suitable for measuring the narrowband or monochromatic light in the region of visible light or a luminescent sensor such as a photomultiplier tube (PMT) sensor as a detector. Recently, mechanoluminescence (ML) has also received much attention because visible light can be directly emitted from micro particles in response to mechanical stimuli such as friction, tension, fracture, and compression [8,9,10,11]. Although CaZnOS crystals can emit ML light with the wavelength ranging from violet to near infrared [11], typically, the ML microparticle emits the monochromatic visible light while exciting or loading, and the spectral information can be changed by the different level of mechanical stimuli. For example, a green phosphor, SrAl2O4:Eu,Dy (SAO), emits the green ML across the region of 500 nm. The potential of ML phenomenon has been successfully expanded into many engineering applications such as sensors, energy harvesting and artificial cochlea [8,9,10,11,12,13,14]. Kim et al. expanded the potential of ML microparticles by providing a preliminary experimental investigation on ML induced by torque and frequency-dependent strain rate [10,12]. Similar to spectrophotometers, a standalone type optomechanical (e.g., pressure into light) sensory system can be then developed based on ML and the miniature spectrometer. The working principle can be described as follows: the visible monochromatic light emission induced by mechanical stimuli is directly measured by using a miniature spectrometer. Therefore, only the miniature spectrometer is required to detect the variation of spectrum signature proportional to the mechanical stimuli (e.g., pressure or stress). 

**Figure 1:**
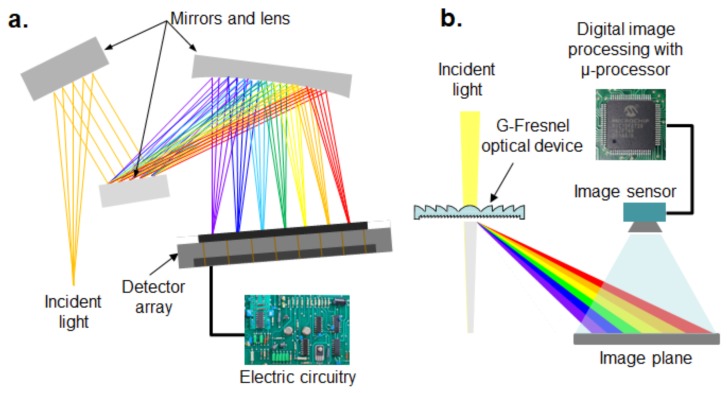
Comparison of the optical spectrometers: (**a**) conventional, (**b**) proposed.

However, although spectrometers play a key role in both spectrophotometry and ML-based optomechanical sensory systems for measuring and analyzing monochromatic light, most conventional commercial spectrometers consist of expensive and bulky optical mirrors, lenses, special electric circuits, and a charge-coupled device (CCD)-type detector array to convert into voltage, resulting in a large detector to capture the broad image plane, as shown in Figure 1a. Furthermore, conventional spectrometers consist of many optical mirrors and lenses to decrease the path of light. To date, few research efforts have been attempted to overcome these technical issues. A Fresnel diffraction-based miniature spectrometer using a Fresnel zone plate and a CCD-based electronic circuit has been developed by Park et al. [15], and they mathematically simulated the linear Fresnel spectrometer [16]. The optical filter array was used instead of a diffraction grating to resolve the input light limitation at specified wavelengths and an algorithm to reconstruct the spectrum [17]. To overcome the resolution issue, the etalon array was used instead of an optical filter array, but the reconstruction algorithm was too complex [18]. Some researchers have attempted to develop spectrometers that mainly focus on the intensity calculation from the spectral image by using image processing software [19]. On the other hand, smartphone-based spectrometers have been recently developed using G-Fresnel optical devices (i.e., diffraction grating and Fresnel lens) to minimize the size. These can also be applied for use as biochemical sensors [20,21]. The G-Fresnel optical device was made of polydimethylsiloxane (PDMS) as an alternative lens material [22,23,24,25,26,27,28]. However, these researchers only focused on the fabrication method of a PDMS-based G-Fresnel lens.

Therefore, the primary objective of this study is to develop a miniature spectrometer that could be used for measuring the wavelength of monochromatic light over the range of visible light. The conventional optical spectrometer used as a detection unit is miniaturized with the developed G-Fresnel grating device in this study, as shown in Figure 1b. The PDMS-based G-Fresnel optical device and new digital image processing algorithm embedded on a μ-processor platform were combined for the standalone type miniature spectrometer.

## 2. G-Fresnel Optical Device and Image Processing Based Miniature Spectrometer

### 2.1. PDMS-Based G-Fresnel Optical Device

**Figure 2:**
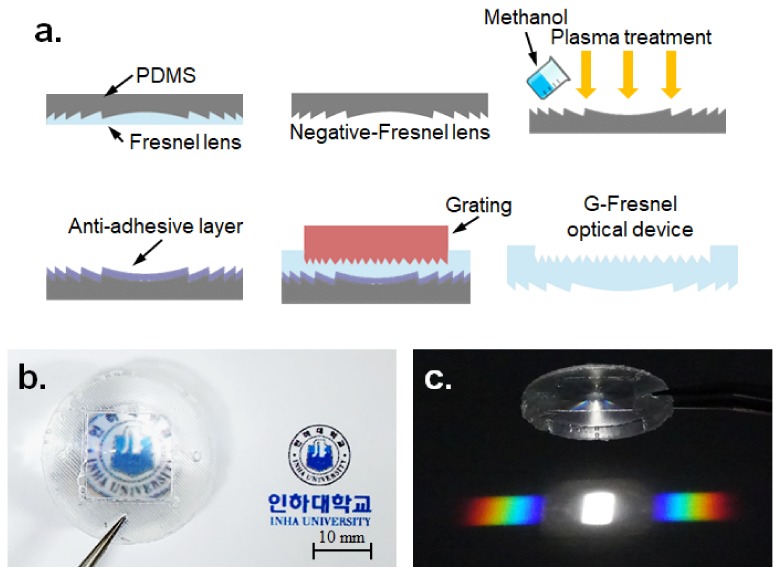
The G-Fresnel optical device. (**a**) Schematic of the manufacturing process, (**b**) and (**c**) Photograph and spectrum of a white light emitting diode (LED). PDMS = polydimethylsiloxane.

**Figure 3:**
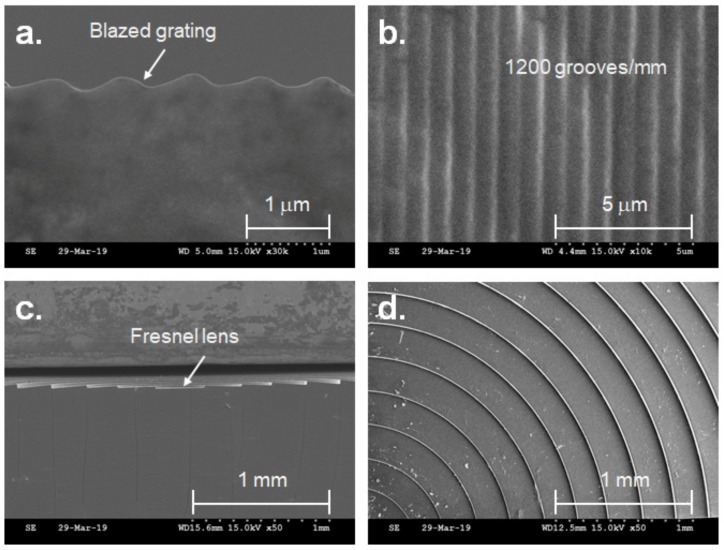
Microscopic scanning electron microscopy (SEM) image of the G-Fresnel optical device. (**a**) Cross section of grating, (**b**) top view of grating, (**c**) cross section of Fresnel, (**d**) top view of G-Fresnel.

The fabrication process for the G-Fresnel optical device is shown in Figure 2a. A mixture of PDMS pre-polymer with a curing agent (Sylgard-184 PDMS, weight ratio 10:1) was poured onto a commercial Fresnel lens (Edmund Optics, 32-588) and cured at low temperature (60 °C) for a day to make a negative Fresnel PDMS lens. Next, oxygen plasma treatment (100 W) was applied to the surface of the negative Fresnel PDMS lens for one minute. Then, methanol was poured on the surface of the negative Fresnel PDMS lens until fully submerged and dried at 80 °C for 30 minutes to generate an anti-adhesive layer [29]; this was done because the PDMS pre-polymer was subjected to bonding with the negative Fresnel PDMS lens in the next molding step. Subsequently, a sandwich molding process was performed to generate the G-Fresnel optical device between the negative Fresnel PDMS lens and the diffraction grating (Edmund Optics, 49-578). The sandwiched PDMS mixture was cured for one day at 60 °C. Finally, the G-Fresnel optical device (approximately Φ = 25 mm) was fabricated by separating the negative Fresnel PDMS lens and the diffraction grating. The G-Fresnel optical device has two functions: a Fresnel lens for focusing and a grating plate for diffraction; these are shown in Figure 2b,c, respectively. A scanning electron microscope (SEM) image of the G-Fresnel optical device is shown in Figure 3. Both the Fresnel lens and diffraction grating surface were successfully, clearly, and sharply transferred.

**Figure 4:**
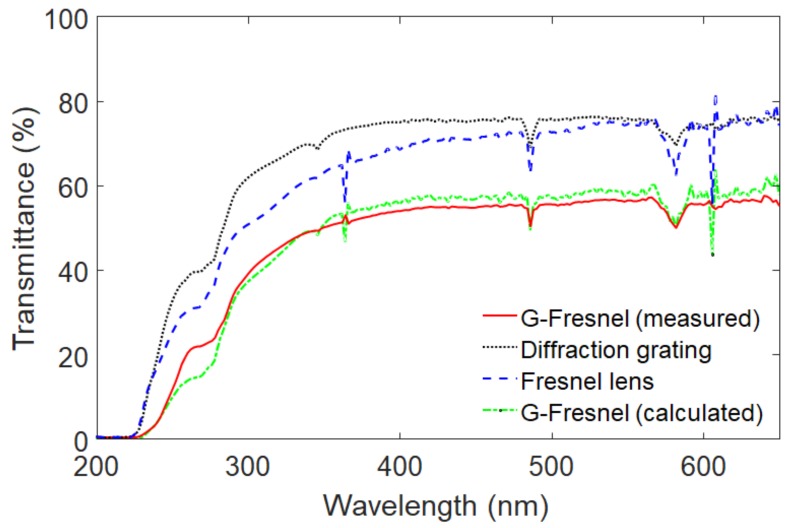
Transmittance curves of the G-Fresnel optical device, a PDMS Fresnel lens, and a PDMS diffraction grating.

The optical transmittance of the G-Fresnel device was examined by a spectrophotometer (Hewlett Packard, 8452A) as shown in Figure 4. The transmittance of the prepared G-Fresnel optical device is approximately 50% in the range of visible light. The transmittance can also be mathematically determined using Equation (1) [30]:

(1) $$T\left(x,y\right)=T_{G}\left(x,y\right)T_{F}\left(x,y\right),$$

where $T\left(x,y\right)$ is the transmittance of the G-Fresnel optical device, $T_{G}\left(x,y\right)$ is the transmittance of the diffraction grating made of PDMS, and $T_{F}\left(x,y\right)$ is the transmittance of the Fresnel lens made of PDMS. The measured transmittance performance of the prepared G-Fresnel optical device shows good agreement with the calculated transmittance from Equation (1) in the range of visible light (i.e., 380–740 nm). 

### 2.2. Design of Miniature Spectrometer

**Figure 5:**
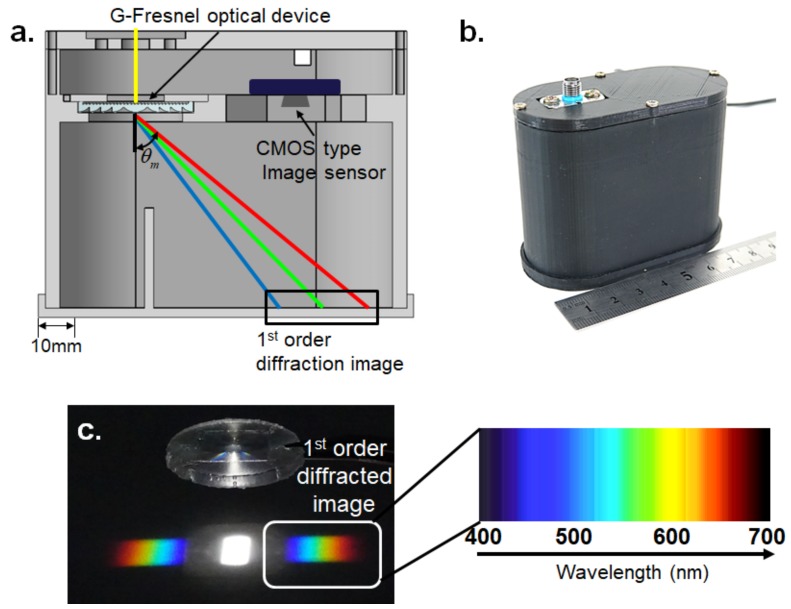
G-Fresnel optical device-based miniature spectrometer (**a**) schematic, (**b**) photograph, and (**c**) the G-Fresnel optical device and spectrum corresponding to white visible light.

The diffracted angle of light should be calculated from the grating equation shown in Equation (2) to design a G-Fresnel optical device-based spectrometer [31]:

(2) $$\theta_{m}=\arcsin\left(\sin\theta_{i}-\frac{m\lambda }{d}\right),$$

where $\theta_{m}$ is the diffracted angle, $\theta_{i}$ is the incidence angle, m is the diffraction order, λ is the wavelength of light, and d is the groove density. Typically, the diffracted angle is affected by the wavelength of light and the length between the lines of grating. In this study, a diffraction grating with a groove density of 1200 grooves/mm was used. Consequently, the diffracted angle is approximately from 28.7° (λ = 400 nm) to 57.1° (λ = 700 nm). As shown in Figure 5a, the slit where the passage of light enters is located on the top of the miniature spectrometer. The spectrum of the light source is produced on the bottom of the miniature spectrometer, and it can be captured by using the CMOS-type image sensor located next to the G-Fresnel optical device. An example of a spectral image for white visible light is shown in Figure 5c. The prototype miniature spectrometer was made by using a three-dimensional (3D) printer and is shown in Figure 5b. The size of the prototype miniature spectrometer is approximately 80 mm (length) × 40 mm (width) × 70 mm (height). Its size is slightly smaller than one of the smallest commercial spectrometers (Ocean Optics, USB-650, Largo, FL, USA), which has a size of 94 mm (length) × 64 mm (width) × 35 mm (height).

### 2.3. Digital Image Processing

**Figure 6:**
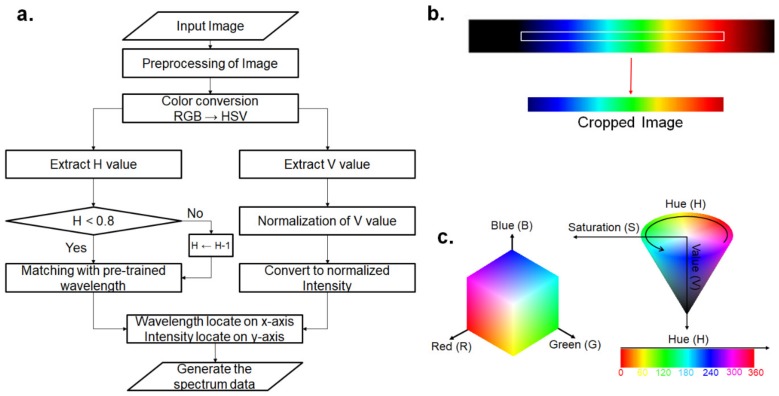
Image processing algorithm: (**a**) flow chart and (**b**) cropped image, (**c**) RGB vs. the hue (*H*), saturation (*S*), and value (*V*) color space.

To miniaturize the spectrophotometer, digital image processing has to be used because it does not require any additional electronic circuitry for processing the output voltage signal from the image sensors. The spectral image captured by the proposed spectrometer should be converted into the wavelength versus intensity relationship by a digital image processing algorithm. A flow chart of the digital image processing algorithm is shown in Figure 6a. First, the image is binarized by Otsu’s binarization for the edge detection algorithm, such as for Canny edge detection. The edge-detected image is used to contour the spectral image, and the region of interest (ROI) is cropped (see Figure 6b). The RGB color space of the cropped image is converted into the hue (*H*), saturation (*S*), and value (*V*) color space, which is one of the generally used color expression methods. This is because the HSV color space represents the dominant color of visible light as just one value or hue (*H*). Moreover, the *V* represents the brightness of each color expressed by the *H*. Therefore, the HSV color space is suitable for acquiring the relationship between the wavelength and intensity from the spectral image of visible light. However, the RGB color space represents the brightness and chromaticity using three variables; therefore, acquiring the relationship between the wavelength and intensity is difficult using the RGB color space. The comparison of the RGB and HSV color space is shown in Figure 6c. The conversion processing from RGB to the HSV color space is represented in Equations (3) and (4) [32]:

(3) $$\begin{array}{l} H=\left\{\begin{array}{cc} \frac{G-B}{\Delta }÷6 & \mathrm{if}\;m_{\max}=R\\ \left(2+\frac{B-R}{\Delta }\right)÷6 & \mathrm{if}\;m_{\max}=G\\ \left(4+\frac{R-G}{\Delta }\right)÷6 & \mathrm{if}\;m_{\max}=B \end{array}\right.\\ H=\begin{array}{cc} H+1 & \mathrm{if}\;H<0 \end{array} \end{array},$$

where $m_{\max}$ is the maximum value among R, G and B of the image pixel, $m_{\min}$ is the minimum value among R, G, and B of the image pixel, and Δ is the value obtained by subtracting $m_{\min}$ from $m_{\max}$. If *H* is negative, it is converted to a positive value by subtracting 1. This is because the color value generally has a positive value.

(4) $$S=\frac{\Delta }{m_{\max}},V=m_{\max}.$$

The ranges for *H* were modified from −0.2 to 0.8 because the *H* could be jumping up around the red color (*H* = 0), whereas *S* and *V* are typically in the range of 0 to 1 according to Equations (3) and (4). Next, the *H* of the spectrum of visible light is obtained continuously without jumping up around the red color. Subsequently, *H* is calibrated with a pre-trained wavelength and *V* is normalized to express the relative intensity of each wavelength. Finally, the calibrated wavelength and normalized intensity are located on the *x*-axis and *y*-axis, respectively, to generate the spectral data of the spectral image.

**Figure 7:**
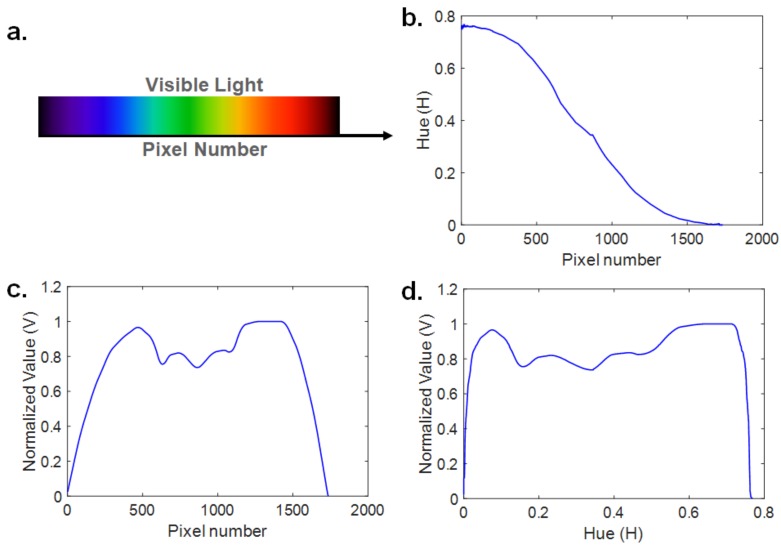
Simulation results from the spectral image of visible light [33]: (**a**) visible spectral image, (**b**) *H* vs. pixel number, (**c**) normalized value (*V*) vs. pixel number, and (**d**) spectrum curve (*V* vs. *H*).

The simulation result of the image processing algorithm is shown in Figure 7. The image used for the simulation is the visible light spectral image shown in Figure 7a [33]. The *H* is decreased nonlinearly like a sigmoid function when the color is changed from violet to red as shown in Figure 7b. This is because the *H* changes very slightly when the wavelength of visible light is around 400 nm and 700 nm. The normalized value, which shows the relative intensity of each wavelength, is shown in Figure 7c. The spectrum data is acquired by locating the *H*, which represents the wavelength of visible light on the *x*-axis, and the normalized value, which represents the relative intensity on the *y*-axis as shown in Figure 7d.

### 2.4. Calibration Results 

**Figure 8:**
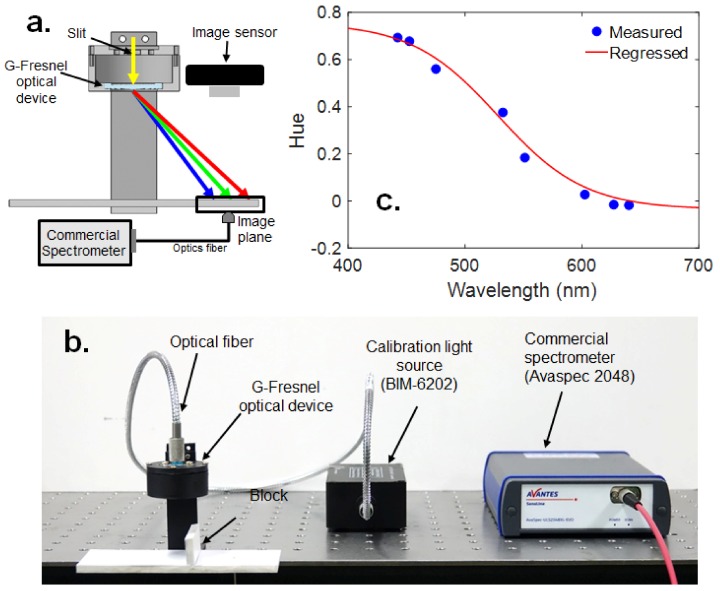
Experimental set-up for calibration: (**a**) schematic, (**b**) photograph, and (**c**) sensitivity curve for calibrating the relationship between wavelength and *H*.

The calibration experiment is performed to identify the relationship between H and wavelength by using the setup shown in Figure 8c. This calibration is performed in a room where it can be completely dark (e.g., at night) to eliminate the effect of background light. The Tungsten light calibration source (Brolight, BIM 6202, Hangzhou, China) is emitted through the optics fiber and passes through the G-Fresnel optical device. Then, the incident light is dispersed and the spectral image is located on the flat image plane (Figure 8a). The spectral image of the calibration source is then captured by the CMOS image sensor and the wavelength of the spectrum is measured at a specific location using a commercial benchtop spectrometer (Avantes, Avaspec-2048, Seoul, Korea). The block is installed between the first-order and zero-order spectrum to block the reflection of the zero-order light. The spectral image of a white light emitting diode (LED) is shown in Figure 8b. The wavelength of the spectrum is measured at eight points by using a commercial spectrometer, and then the *H* of the spectrum, which is located on certain points (from 1–8), is measured simultaneously.

The relationship of *H* versus the wavelength of light was obtained from the calibration experiment, as shown in Figure 8c, and identified by a sigmoid nonlinear function similar to the simulation (i.e., Figure 7b). The mathematical equation for the sensitivity curve can be fitted by the following regression formula (Equation (5)):

(5) $$y=0.7556+\left(\frac{-0.7926}{1+e^{\frac{528.4529-x}{37}}}\right),$$

where *y* is the *H* of the position where the wavelength is measured, and *x* is the wavelength of the spectrum at a certain location.

**Figure 9:**
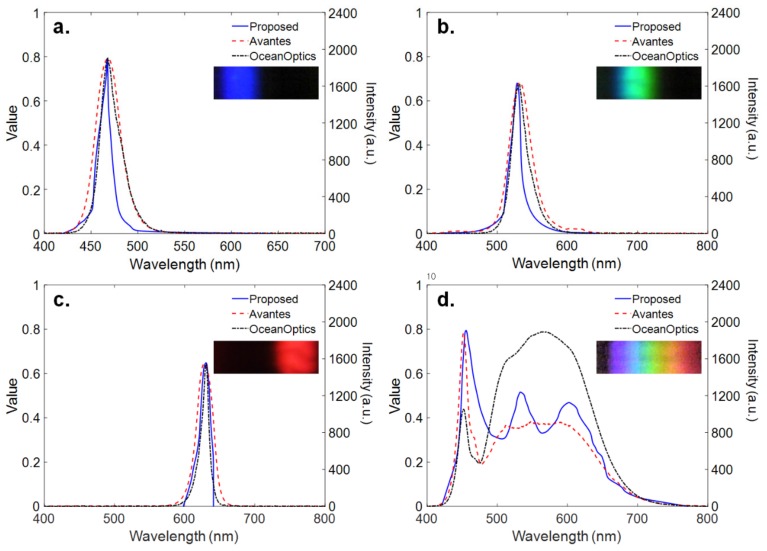
Spectrum for different visible light sources (**a**) blue, (**b**) green, (**c**) red, and (**d**) white. For more information, please refer to the Supplementary Materials.

Finally, narrow-band LEDs for various visible light sources were used to evaluate the performance of the proposed miniature spectrometer and compared with the results by using two benchtop commercial spectrometers (Avantes, Avaspec-2048, and Ocean Optics, USB650). This experiment was performed using a PC and the MATLAB/Image Processing Toolbox provided by MathWorks^TM^ [34]. As shown in Figure 9a, the wavelength where the maximum intensity is measured by the proposed spectrophotometer peaks at 470 nm. As shown in Figure 9b,c, the wavelength distribution of the green and red LED is shifted compared to the blue LED, and peaks at approximately 520 nm and 630 nm, respectively. The difference from the results using two commercial spectrometers ranges from 1 nm to 3 nm. The spectrum of a white LED exhibits the peak at about 465 nm due to the blue light directly emitted by the GaN-based LED and the broadband spectrum emitted by the phosphors, as shown in Figure 9d. Note that three spectra measured by the proposed and two commercial spectrometers show the different spectra over the range from 500 to 700 nm. However, the proposed spectrometer shows good agreement with the commercial products at the peak of 465 nm with an error of 3 nm. Overall, the spectrum measured by using the proposed miniature spectrophotometer showed a good agreement with the two spectra measured by using two current commercial spectrometers, which implies reliable performance for measuring the spectrum information of visible light, particularly monochromatic light. Similar to a detector of the spectrophotometer, the proposed miniature spectrometer is used for measuring the wavelength of monochromatic light.

## 3. Application to Mechanoluminescent Optomechanical Sensors

**Figure 10:**
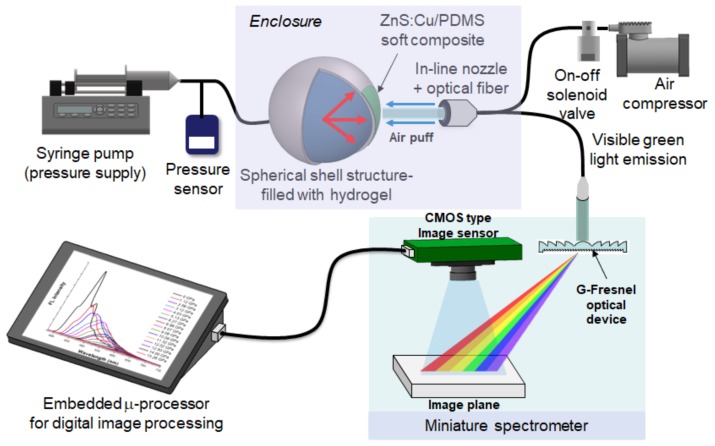
Schematic of the standalone opto-mechanical sensory system based on mechano-luminescence and the miniature spectrometer.

**Figure 11:**
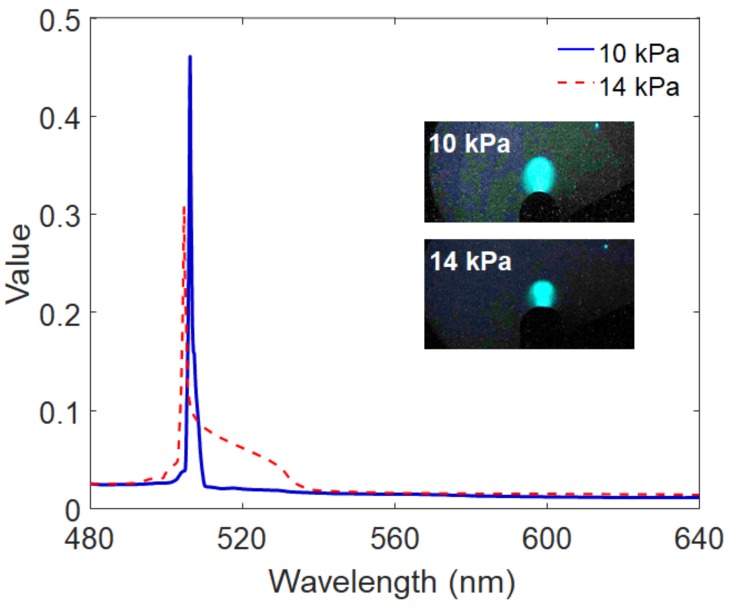
Example of spectra illustrating the intraocular pressure (IOP) monitoring principle based on a mechanoluminescent optomechanical sensory system (inset: captured ML images for different IOPs).

This miniature spectrometer can be applied to mechanoluminescence (ML) optomechanical sensory systems (in this study, intraocular pressure (IOP) monitoring). IOP can be indirectly monitored by measuring the wavelength or intensity of ML emitted from an ML composite membrane attached to the fluid-filled spherical polymer structure representing our human vision system (the eye). ML induced by air-puff excitation is measured inside of an enclosure (i.e., dark environment) for sensitive optical recording and blocking background light. As shown in Figure 10, the ZnS:Cu/PDMS soft composite emits the ML of green light across the region of 500 nm when external pressure is applied by periodic air-puff actuation, which is identical to air-puff type tonometers. The spectral information of ML is in turn analyzed by the miniature spectrometer, and provides the variation signature of IOP monitoring, as shown in Figure 11. The spectral image can be captured by this optomechanical sensory system either in real time (online) or offline (i.e, stored) due to the digital image processing, whereas the conventional PMT sensor or spectrometer can only measure instantaneous light. The offline operation will induce the amplification effect of intensity when the ML image is captured by a high-speed camera. The images per frame can be accumulated and eventually amplified in a similar way to PMT sensors. 

## 4. Conclusions

In this study, we demonstrated a G-Fresnel optical device-based miniature spectrometer for wavelength and relative intensity measurements of visible light, such as an LED light source by the HSV color space, to yield a nonlinear sensitivity (*H* vs. the wavelength). In comparison to Fraunhofer diffraction-based commercial benchtop spectrometers, the proposed miniature spectrometer offers a very compactly sized package owing to the G-Fresnel optical device and simple digital image processing algorithm. By virtue of its compact size and cost-effectiveness, this instrument may be an attractive solution for the indirect measurement of physical quantities, such as pressure, by estimating the wavelength proportional to physical quantities because: (a) only a cost-effective CMOS image sensor is required to detect the wavelength and intensity of the visible light and (b) this miniature spectrometer does not require any complicated electronic circuits or optical theories. To the best of our knowledge, we report for the first time that it is possible to achieve a new means of determining the wavelength information from the spectra based on a G-Fresnel optical device and digital image processing by the HSV color space. Because this spectrometer has the potential for an opto-mechanical monitoring system, where the wavelength is directly proportional to the physical quantities such as pressure and strain rates, these conservative results are sufficient to demonstrate the effectiveness of the proposed miniature spectrometer for measuring monochromatic light such as the mechanoluminescence of green light across the region of 500 nm. Further studies will focus on the implementation of an embedded μ-processor for real-time measurement, further miniaturization and improvements in the image processing algorithm of the G-Fresnel optical device.

## Supplementary Materials

The following are available online at https://www.mdpi.com/1424-8220/19/16/3528/s1, Video S1: Miniature Spectrometer based on a G-Fresnel Optical Device and Image Processing by HSV Color Space.
